# The downside of choice: Having a choice benefits enjoyment, but at a cost to efficiency and time in visual search

**DOI:** 10.3758/s13414-016-1062-2

**Published:** 2016-02-18

**Authors:** Melina A. Kunar, Surani Ariyabandu, Zaffran Jami

**Affiliations:** Department of Psychology, University of Warwick, Coventry, CV4 7AL UK

**Keywords:** Visual search, Cognitive and attentional control, Decision making

## Abstract

The efficiency of how people search for an item in visual search has, traditionally, been thought to depend on bottom-up or top-down guidance cues. However, recent research has shown that the rate at which people visually search through a display is also affected by cognitive strategies. In this study, we investigated the role of choice in visual search, by asking whether giving people a choice alters both preference for a cognitively neutral task and search behavior. Two visual search conditions were examined: one in which participants were given a choice of visual search task (the choice condition), and one in which participants did not have a choice (the no-choice condition). The results showed that the participants in the choice condition rated the task as both more enjoyable and likeable than did the participants in the no-choice condition. However, despite their preferences, actual search performance was slower and less efficient in the choice condition than in the no-choice condition (Exp. 1). Experiment 2 showed that the difference in search performance between the choice and no-choice conditions disappeared when central executive processes became occupied with a task-switching task. These data concur with a *choice-impaired* hypothesis of search, in which having a choice leads to more motivated, active search involving executive processes.

People search for objects regularly in everyday life. Scientists study these processes in the laboratory by asking participants to search for a target among distractors and recording accuracy and reaction times (RTs). Search efficiency is determined by the RT × Set Size function, which measures how RTs increase with the number of items (set size), to give the *search slope* (Treisman & Gelade, 1980). The shallower the search slope, the more efficient the search task. Previous research has suggested that the rate at which we search is largely determined by the type of stimuli present (e.g., Duncan & Humphreys, 1989; Treisman & Gelade, 1980; Wolfe, Cave, & Franzel, 1989). Search efficiency can depend on bottom-up processes, in which salient stimuli attract people’s attention, or top-down processes, in which prior knowledge of the target’s features guide attention to the stimuli that are task-relevant (see Wolfe & Horowitz, 2004, for a review).

Recent work, however, has suggested that cognitive strategies may also influence how people search a display. For example, Smilek, Enns, Eastwood, and Merikle (2006) found that search efficiency was influenced by the type of search strategy people adopted. Participants were given a difficult visual search task and asked to search for the target either actively (e.g., to direct their attention deliberately to items in the display) or passively (e.g., to let their intuition guide their response). Even though the stimuli were the same in both conditions, the results showed that search slopes in the passive condition were more efficient than those in the active condition (see also Kunar et al., 2014, and Lleras & Von Mühlenen, 2004, who showed that different strategies affect visual search). Passive viewing also resulted in fewer eye movements to find the target than when participants actively viewed the display (Watson, Brennan, Kingstone, & Enns, 2010), and reduced the deficit witnessed in the attentional blink (Olivers & Nieuwenhuis, 2005). Given that these attentional tasks have been influenced by differing cognitive strategies, it is possible that other types of contexts would also have an effect on cognitive decisions. We examined this here by looking at the effect of choice on visual search.

Much research has shown that the role of choice has a strong influence on people’s preferences and motivation (e.g., Bown, Read, & Summers, 2003). For example, giving people a choice has led to better performance in education (Reynolds & Symons, 2001), increased levels of creativity (Amabile & Gitomer, 1984), and improved well-being in elderly patients (Schulz, 1976). The appeal of choice is so strong that people often choose options that give them a wider set of choices, even when the increased number of options does not necessarily improve utility (Bown et al., 2003). Furthermore, people show a tendency to keep their options open and preserve their choice, even if this means losing out on monetary payment (Shin & Ariely, 2004).

There are several explanations for why people prefer having a choice rather than no choice at all. Bown et al. (2003) argued that people do not initially weigh the options given in front of them, but instead adopt a simplified heuristic that it is better to have a larger set of options than a smaller one. This makes sense in evolutionary terms; for example, our ancestors would have learned that a land with more species to hunt is much better than a land with fewer (Bown et al., 2003). A more biological explanation is that people find the opportunity to make a choice desirable because it increases activity in the ventral striatum, which is part of the brain circuitry thought to be involved in reward processing and motivating behavior (Leotti & Delgado, 2011). This may be related to the idea that people feel a greater sense of control when given a choice (Inesi, Botti, Dubois, Rucker, & Galinsky, 2011; Langer, 1975), although Leotti and Delgado argued that the opportunity to choose, in itself, is inherently valuable. Whatever the explanation, it is clear that, given the option, people prefer to have a choice rather than no choice at all.

Although previous research has shown that people prefer choice, this has been demonstrated using questions that typically have meaning for the participant (e.g., would you prefer outcome A or outcome B?). In this study, we tested whether having a choice in itself is inherently valuable, by using a cognitively neutral task of little personal value to the participant. Here we gave participants a visual search task, asking them to search for an affectively neutral target (the letter T) among affectively neutral distractors (letter Ls). We used these stimuli to ensure that nothing was inherent in the task for people to either like or dislike (typically in tasks like these, participants may, if anything, find the task tedious). Two conditions were tested: a “choice” condition, in which we gave people a choice of which visual search task they could take part in (Condition A, B, or C), and a “no-choice” condition, in which people were assigned to a predetermined visual search task. Importantly, unbeknownst to participants in the choice condition, all three visual search tasks were exactly the same (search for a T among Ls). Following Experiment 1, participants were asked to rate how much they enjoyed the task, how much they liked the task, and how difficult they found the task overall.

Our study addressed two questions. First, would giving people a choice increase their preference of a cognitively neutral task? If so, there should be a marked increase of enjoyment and liking of the task in the choice relative to the no-choice condition. Second, would giving people a choice lead to a change in visual search performance? There were two possibilities. First, if the rate of visual search is largely determined by bottom-up and/or top-down strategies (e.g., Treisman & Gelade, 1980), then giving participants a choice should not affect search efficiency at all. We call this the *choice-neutral* hypothesis. In contrast, giving people a choice could lead them to have increased motivation in the task and to adopt a more active role when searching. It is well-documented that people engage and persist more in a task if they have chosen it (e.g., Deci, 1980; Lewin, 1952; Patall, Cooper, & Robinson, 2008; Ryan & Deci, 2000). This has implications for search: According to Smilek et al. (2006), if people adopt an active strategy, their executive processes become involved in the task, which leads to less efficient search behavior. Therefore, if having a choice results in people adopting a more active, motivated strategy, then search should be less efficient. We call this the *choice-impaired* hypothesis. The results of Experiment 1 showed that although giving participants a choice enhanced their enjoyment of the search task, their performance in relation to both search times and search efficiency worsened. In Experiment 2, we investigated whether central executive processes were needed for this effect to occur. If so, when we ensured that executive processes were already occupied with a task-switching task,^1^ the difference in search performance between conditions should disappear. These results showed that when the executive processes were fully utilized in both conditions, there was no additional effect of choice on search performance.

## Method

### Participants

In all, 100 participants (61 female, 39 male; mean age = 21.0 years) took part in Experiment 1, and 20 participants took part in Experiment 2^2^ (11 female, nine male; mean age = 25.7 years). All of the participants had normal or corrected-to-normal vision. In each experiment, participants completed either the choice or the no-choice condition (with equal numbers of participants assigned to the two conditions).

### Apparatus and stimuli

Displays were generated and responses recorded using Blitz 3-D programs. The distractors were L shapes presented randomly in one of four orientations (0°, 90°, 180°, or 270°) on a black background. In Experiment 1, the target was a T, rotated 90° or 270° with equal probabilities, and in Experiment 2, the target was a T, rotated 0° or 180° with equal probabilities. Each L contained a small offset (~0.5°) at the line junction to make search more difficult. All stimuli subtended 1.7° × 1.7°, at a distance of 57.4 cm. Each display had a set size of 4, 8, or 12 items, with individual stimuli positioned within a 6 × 6 invisible matrix. Each display was uniquely generated, and therefore displays were not repeated across the experiment. In Experiment 1, all of the stimuli were white. In Experiment 2, on half of the trials all stimuli were red, and on the other half all stimuli were green.

### Procedure

**Fig. 1:**
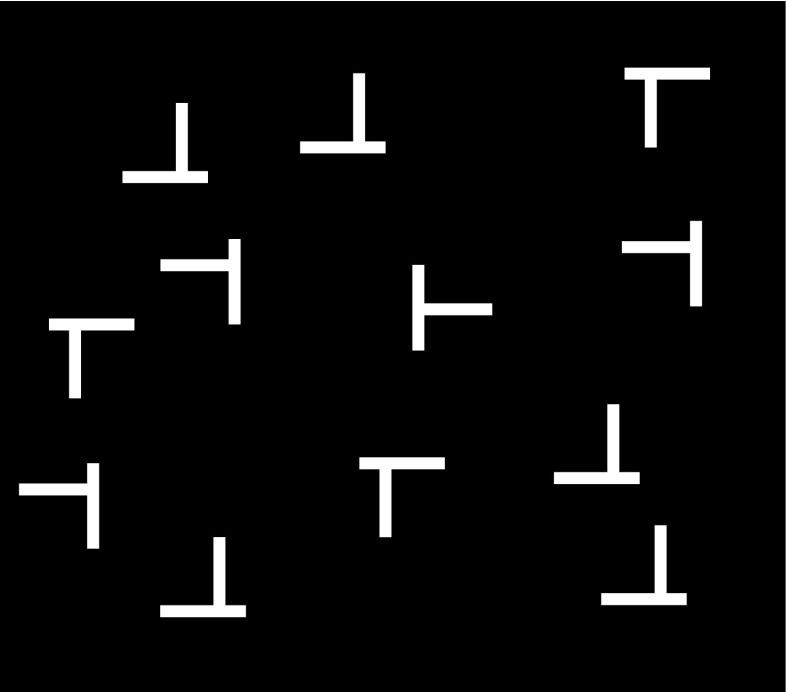
Example of the visual search display used in Experiment 1. The target is a T among rotated Ls.

Participants completed an experimental block of 90 trials (30 for each set size). On each trial, a fixation dot was presented for 500 ms, followed by the distractors and target letter. The stimuli were presented on the screen until participants made a response. If participants had not responded in 10,000 ms, the display timed out and the next trial was presented. Participants were asked to respond as quickly and as accurately as possible, and completed a short practice session before each block. Example displays are shown in Fig. 1. In Experiment 1, participants were asked to search and respond to the orientation of the T, by pressing “m” if the bottom of the T pointed to the right or “z” if the bottom pointed to the left. In Experiment 2, the task changed on different trials, depending on the color of the stimuli. If the stimuli were red, participants were asked to search for an upright T; if the stimuli were green, they were asked to search for an inverted T. Participants pressed “m” if the target was present and “z” if the target was absent.

In the choice conditions, participants were asked to choose which experimental block they would like to complete. Their choice options were that they could complete either “Condition A,” “Condition B,” or “Condition C.” No other information about the blocks was given to participants. Once participants had chosen, they were given the corresponding condition. However, unknown to the participants, all conditions were the same in every way but their name. In the no-choice conditions, participants were told they had been assigned to one of the conditions and were not given a choice. In Experiment 1, participants were asked to rate how much they enjoyed the task, how much they liked the task, and how difficult they found the task. Responses were collected using three Likert scale questions ranging from 1 (*not at all*) to 7 (*very much*).

## Results

**Fig. 2:**
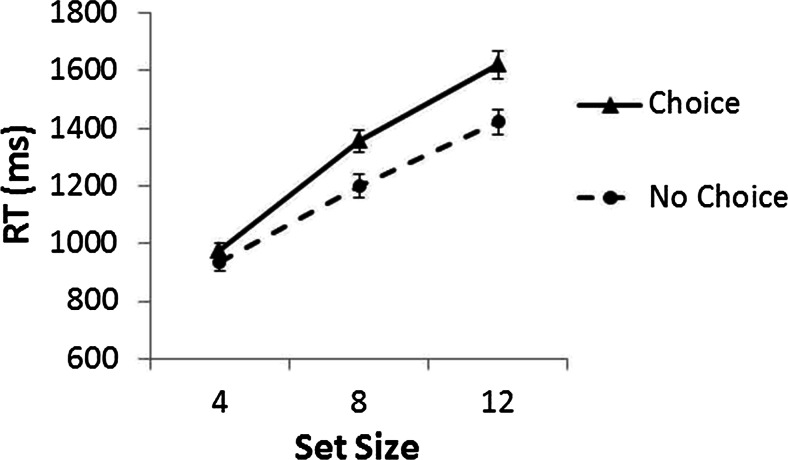
Mean correct reaction times (RTs, in milliseconds) across set sizes for the choice and no-choice conditions in Experiment 1. Error bars represent the standard errors.

**Fig. 3:**
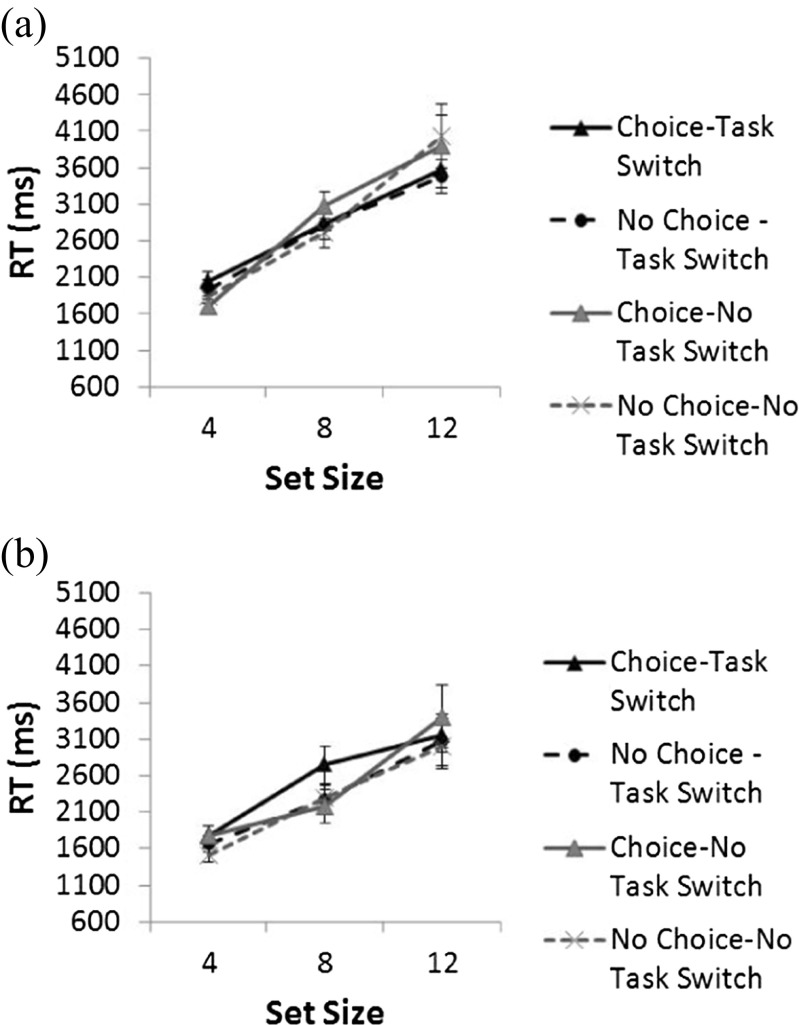
(a) Mean correct reaction times (RTs, in milliseconds) across set sizes for all conditions in Experiment 2, for target-absent trials. (b) Mean correct RTs (in milliseconds) across set sizes for all conditions in Experiment 2, for target-present trials. Error bars represent the standard errors.

In Experiment 1, RTs less than 200 ms and greater than 4,000 ms were removed as outliers. This led to the removal of 1.4 % and 1.7 % of the data from the choice and no-choice conditions, respectively. RTs less than 200 ms were also removed from Experiment 2 (0 % of the data); however, because the task was more difficult, no upper outlier boundary was applied.^3^ Mean correct RTs are shown in Figs. 2 and 3. For Experiment 1, a mixed-design 2 (choice vs. no choice) × 3 (set size: 4, 8, or 12) analysis of variance (ANOVA) on correct participant mean RTs was conducted, with the within-participants factor Set Size and the between-participants factor Choice. The results showed a main effect of choice, *F*(1, 98) = 7.2, *p* < .01, *η*_p_^2^ = .068, in which RTs in the choice condition (1,318 ms) were slower than those in the no-choice condition (1,186 ms), and a main effect of set size, *F*(2, 196) = 449.2, *p* < .01, *η*_p_^2^ = .821, in which RTs increased with set size. The Choice × Set Size interaction was also significant, *F*(2, 196) = 9.0, *p* < .01, *η*_p_^2^ = .084. RTs increased more with set size, showing less efficient search, in the choice condition (80.7 ms/item) than in the no-choice condition (61.2 ms/item).

However, this difference disappeared in Experiment 2, with the engagement of executive processes in the task-switching task. Here, a mixed-design 2 (choice vs. no choice) × 3 (set size: 4, 8, or 12) × 2 (task switch vs. no task switch) × 2 (present vs. absent) ANOVA on correct participant mean RTs was conducted, with the within-participants factors Set Size, Task Switch, and Target Presence, and the between-participants factor Choice. We observed main effects of target presence, *F*(1, 18) = 25.7, *p* < .01, *η*_p_^2^ = .588, and of set size, *F*(2, 36) = 112.4, *p* < .01, *η*_p_^2^ = .862. RTs for target-present trials were faster than target-absent ones, and RTs increased with set size. However, we found no main effect of task switch (*F* < 1), nor a main effect of choice (*F* < 1). A significant Target Presence × Set Size interaction was apparent, *F*(2, 36) = 3.2, *p* = .05, *η*_p_^2^ = .151, in which RTs increased more with set size for target-absent than for target-present trials, as well as a significant Task Switch × Set Size interaction, *F*(2, 36) = 3.8, *p* < .05, *η*_p_^2^ = .174, in which RTs increased more with set size for no-task-switch than for task-switch trials. The four-way Target Presence × Task Switch × Set Size × Choice interaction was also significant, *F*(2, 36) = 3.2, *p* = .05, *η*_p_^2^ = .150. None of the other interactions were significant. Error rates are shown in Table 1. For Experiment 2, we observed a main effect of target presence, *F*(1, 18) = 8.6, *p* = .01, *η*_p_^2^ = .309, in which more errors occurred on target-present trials (4.04 %) than on target-absent trials (4.02 %), and a main effect of set size, in which errors increased with set size, *F*(2, 36) = 4.0, *p* < .05, *η*_p_^2^ = .181. However, none of the other main effects or interactions in either experiments were significant.

**Table 1:** Percentages of errors for the choice and no-choice conditions across set sizes

	Set Size		
Condition	4	8	12
Experiment 1, choice	1.9	2.3	2.4
Experiment 1, no choice	2.1	2.4	1.9
Experiment 2, choice	3.7	6	8.7
Experiment 2, no choice	5	3	7

**Fig. 4:**
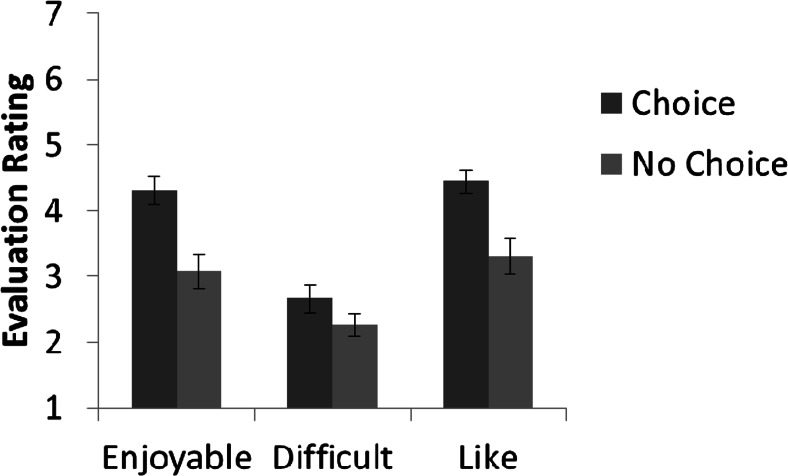
Mean ratings on the Likert scales evaluating how enjoyable participants found the task, how difficult they found the task, and how much they liked the task. The scales ranged from 1 (*not at all*) to 7 (*very much*). Error bars represent the standard errors.

Figure 4 shows the mean ratings from the three Likert scales in Experiment 1, asking participants how much they enjoyed the task, how much they liked the task, and how difficult they found the task. Three separate Mann–Whitney *U* tests showed that participants in the choice condition rated the visual search task to be more enjoyable than did participants in the no-choice task (average rank: choice = 59.7, no choice = 41.3; *U* = 789.0, *p* < .01, *r* = –.32). Furthermore, participants in the choice task showed a more positive rating when asked how much they liked the task, as compared with participants in the no-choice task (average rank: choice = 59.9, no choice = 41.0; *U* = 778.0, *p* < .01, *r* = –.33). However, no differences emerged in ratings of how difficult participants found the task across the choice and no-choice groups (average rank: choice = 53.8, no choice = 47.2; *U* = 1,087.0, *p* = .25, *r* = –.11).

## Discussion

Previous work has shown that people prefer options that give them a choice (e.g., Bown et al., 2003). In this study we examined whether people would rate a cognitively neutral task more positively when they were offered a choice than when no choice was given. The results showed that people had a strong preference for a condition in which they were given a choice, by rating the choice condition as more enjoyable and reporting greater liking of the task. Interestingly, this preference for choice occurred in the absence of any personal meaning to the participants. Participants were not informed of what the different conditions were, and thus were unable to make an informed choice. Furthermore, because the task was devoid of affective and meaningful stimuli, and instead consisted of neutral letter stimuli, there should not have been anything inherently pleasurable about doing the task. Despite this, the mere fact that participants were given a choice led them to value the task more.

However, the option of a choice also had a negative impact on search performance. Search was less efficient in the choice condition than in the no-choice condition (Exp. 1). Furthermore, response speed in the choice condition was impaired relative to the no-choice condition. These results cannot be explained by a speed–accuracy trade-off, since error rates were low and there were no differences in errors for choice across conditions. Instead, the data are consistent with the *choice-impaired* hypothesis, in which giving people a choice changed their cognitive strategy, leading to less efficient search.

Previous research has shown that if participants adopt an active search strategy, they show less efficient search than do those who use a more passive strategy (Smilek et al., 2006). Smilek et al. argued that this occurred because participants were relying on slow executive-control mechanisms when performing an active search. In contrast, people who were passively searching relied on automatic mechanisms that allowed for more rapid search overall (Smilek et al., 2006). It is also well-known that giving people a choice leads to greater motivation and effort in a task (e.g., Patall et al., 2008). This can explain the results in our study, with people adopting a more active search strategy involving executive processes after the act of choosing had led them to be more motivated and invested in the task. The use of executive processes in the choice condition would have led to the reduced search efficiency observed in Experiment 1. Experiment 2 gives weight to this argument, since it shows that when the executive processes were otherwise occupied in the task-switching task, for both the choice and no-choice conditions, the difference in search performance across conditions disappeared. That is, because the executive processes were already being utilized, they would not be available to affect search in the choice condition. One could argue that trials in which the task was the same on trial *n* as on trial *n*–1 (i.e., task-repeat trials) in Experiment 2 were similar to those in Experiment 1, and therefore should have been affected by choice. However, crucially, in Experiment 2 participants were required to maintain the task instructions in working memory throughout the experiment (including trials in which the task was repeated). Therefore, because executive processes were being utilized on all trial types, they would not be available to affect search in the choice condition. The ideas of executive control and automatic mechanisms could be considered similar to people’s dual processing system, commonly known in the field of decision making as System 1 and System 2. *System 1* refers to automatic procedures that are quick, effortless, and occur in parallel, whereas *System 2* requires much more detailed, controlled, and complex cognitive resources (Kahneman, 2003). In our study, we suggest that participants used the slower, more thoughtful System 2 during the choice condition of Experiment 1, resulting in slowed RTs and less efficient search.

The present data show that although presenting people with a choice led to greater enjoyment, it did so at the price of impairing search performance. It is generally considered that having a choice is beneficial (Patall et al., 2008). However, along with the benefits, these data show the costs of choice in terms of time and efficiency. The data also add to the growing evidence that performance in attentional tasks can be altered by cognitive strategies. This may explain some of the recent replication difficulties in the literature (as part of the “replication crisis”; Schooler, 2014). If participants’ cognitive strategies are different across replication attempts, different results may emerge. Further research will be needed to investigate this, but for now the present data highlight an important issue for scientists—namely, that experimental outcomes can be affected by a participant’s frame of mind.

## Footnotes

1 It is well-documented that task switching requires the use of executive processes (see, e.g., Monsell, 2003).

2 Fewer participants were required for Experiment 2 because it did not involve Likert questionnaires. Typical experiments investigating slope differences in visual search tasks have used approximately 5–15 participants (e.g., Duncan & Humphreys, 1989; Treisman & Gelade, 1980; Wolfe, 1998). Therefore, following these examples, we tested 20 participants in Experiment 2 to ensure enough statistical power to detect an effect, if one were present.

3 Please note that the pattern of data for Experiment 2 was the same if the outlier removal procedure of Experiment 1 was used.
